# Stratified therapeutic security and understanding backwards care pathway moves. A 5-year retrospective cohort analysis from the Dundrum Forensic Redevelopment Evaluation (D-FOREST) study in Dublin, Ireland

**DOI:** 10.1192/j.eurpsy.2023.923

**Published:** 2023-07-19

**Authors:** L. Jordan, G. Crudden, D. Mohan, H. Kennedy, M. Davoren

**Affiliations:** 1 National Forensic Mental Health Service, Central Mental Hospital, Dundrum, Dublin, Ireland; 2 Trinity College Dublin; 3 National Forensic Mental Health Service, Central Mental Hospital, Dundrum, Dublin, Ireland and Trinity College Dublin, Dublin, Ireland

## Abstract

**Introduction:**

Secure forensic hospital settings provide care and treatment to mentally disordered offenders with a history of serious violence. Most modern forensic hospitals operate a system of stratified therapeutic security, where patients are placed on the internal care pathway according to individual risks and needs. Unfortunately, at times patients move ‘backwards’ from a unit of lower to a unit with higher therapeutic security. This is a challenge to manage from an individual patient and service perspective.

**Objectives:**

The aim of this study was to analyse backwards moves along the care-pathway within a complete national cohort of forensic in-patients in Ireland over a five-year period. We aimed to clarify the reasons for these moves and ascertain if they were linked to mental illness, security or other issues.

**Methods:**

A naturalistic retrospective five-year observational cohort study was completed. All in-patients in the Central Mental Hospital, Dundrum, Ireland or associated high support hostels between January 2016 and January 2021 were included (60 months). Demographic data, data pertaining to diagnosis, data pertaining to backwards moves and reasons for those moves were gathered. Data was gathered as part of the Dundrum Forensic Redevelopment Evaluation study (D-FOREST study).

**Results:**

A total of n=231 patients were included; the majority (n= 203; 87.9%) were male. The most common diagnosis was schizophrenia (64.1%), followed by schizoaffective disorder (12.6%), bipolar affective disorder (4.8%) and autistic spectrum disorder (3.5%). Mean age at admission was 35.9 years, SD 9.5.

Over the 60-month period, a total of 93 backwards moves relating to 50 patients occurred. Reasons for backward moves included deteriorating mental state (8.7%), assaults (4.3%), challenging behaviour (4.3%), security (1%) and others. Binary logistic regression demonstrated that lacking capacity to consent to medication (Odds ratio 0.352, 95%CI 0.198-0.627, p<0.001) and higher (worse) scores on HCR-20 Historical scale (Odds ratio 1.13, 95%CI 1.01-1.27, p=0.035) were associated with backwards moves, when adjusting for age and Dundrum-1 need for therapeutic security scores.

**Conclusions:**

Backwards care pathway moves are a major issue in forensic hospitals both nationally and internationally. We were surprised at the strength of association between lacking capacity to consent and backwards moves. Understanding backwards moves will assist in supporting patients and minimising length of stay.

**Disclosure of Interest:**

L. Jordan Grant / Research support from: This study was funded by the Health Service Executive for the Republic of Ireland as part of the Dundrum Forensic Redevelopment Evaluation (D-FOREST) study, G. Crudden: None Declared, D. Mohan: None Declared, H. Kennedy: None Declared, M. Davoren: None Declared

